# Cross-feeding of bifidobacteria promotes intestinal homeostasis: a lifelong perspective on the host health

**DOI:** 10.1038/s41522-024-00524-6

**Published:** 2024-06-19

**Authors:** Meifang Xiao, Chuan Zhang, Hui Duan, Arjan Narbad, Jianxin Zhao, Wei Chen, Qixiao Zhai, Leilei Yu, Fengwei Tian

**Affiliations:** 1 State Key Laboratory of Food Science and Resources, Jiangnan University, Wuxi, Jiangsu 214122 P. R. China; 2 School of Food Science and Technology, Jiangnan University, Wuxi, Jiangsu 214122 China; 3 Quadram Institute Bioscience, Norwich Research Park Colney, Norwich, Norfolk, NR4 7UA UK; 4 National Engineering Research Center for Functional Food, Jiangnan University, Wuxi, Jiangsu 214122 China

**Keywords:** Applied microbiology, Microbial communities

## Abstract

Throughout the life span of a host, bifidobacteria have shown superior colonization and glycan abilities. Complex glycans, such as human milk oligosaccharides and plant glycans, that reach the colon are directly internalized by the transport system of bifidobacteria, cleaved into simple structures by extracellular glycosyl hydrolase, and transported to cells for fermentation. The glycan utilization of bifidobacteria introduces cross-feeding activities between bifidobacterial strains and other microbiota, which are influenced by host nutrition and regulate gut homeostasis. This review discusses bifidobacterial glycan utilization strategies, focusing on the cross-feeding involved in bifidobacteria and its potential health benefits. Furthermore, the impact of cross-feeding on the gut trophic niche of bifidobacteria and host health is also highlighted. This review provides novel insights into the interactions between microbe-microbe and host-microbe.

## Introduction

Bifidobacteria are abundant in the human gut and other warm-blooded animals, and are present in the rumen of ruminants, vagina^1^, intestines of honeybees^2^, oral cavity^3^, dairy products^4^, and breast milk^5^. Gut colonization by bifidobacteria occurs in the early stages of life and is closely associated with health and aging. Bifidobacteria are transmitted vertically from mother to offspring and are commonly found in the gut of healthy breastfed infants, accounting for 60–70% of all gut bacteria^6^. Aging alters the number and diversity of bifidobacteria in the human gut. In adulthood, the relative abundance of bifidobacteria decreases to approximately 10%, however remains stable, whereas that in aging individuals accounts for approximately 5% of the total gut bacteria^6^. In infancy, *Bifidobacterium breve*, *B. longum*, and *B. bifidum* are commonly found in feces^7^, while in adulthood, *B. adolescentis*, *B. longum*, and *B. pseudocatenulatum* are the dominant species^8,9^. The relative abundance of *B*. *adolescentis* decreases with age, whereas that of *B*. *breve* and *B. longum* is dominant in the gut of aging individuals^10^.

The rich and complex sources of carbon in the gut tract provide a trophic niche for gut microbiota. The differential utilization of glycans influences gut microbiota formation in gut niches, which may explain why certain species of bifidobacteria are more common in the gut tract of infants or adults^11,12^. Infant breast milk and adult diets contain a high abundance of glycans and oligosaccharides with complex structures that cannot be digested by the human body and hence pass through the large intestine as substrates for the gut microbiota. Bifidobacteria metabolize monosaccharides, disaccharides, and oligosaccharides; however, different bifidobacterial species prefer dietary glycans, including inulin-type fructan (ITF)^13,14^, resistant starch (RS)^15^, galactan^16,17^, xylan^18,19^, and arabinan^20^, while others utilize host-derived glycans, such as human milk oligosaccharides (HMOs)^21,22^ and mucins^23^.

The metabolism of complex glycans by human gut microbiota is mediated by carbohydrate-active enzymes (CAZymes). Bifidobacteria has been estimated to use approximately 14.64% of the CAZyme gene, including glycosyl hydrolases (GHs) and glycosyl transferases (GTs), for the transport, degradation, and regulation of glycans, which is only slightly less than that of *Bacteroides* (20%). Gut microbiota employ similar strategies to maintain and break down complex glycans^24,25^. Bifidobacteria encode a series of modular glucanase complexes that are anchored to the cell surface by transmembrane domains. These contain genes encoding GHs to extracellularly digest long oligosaccharides or glycans and substrate-binding proteins (SBPs) of the adenosine triphosphate (ATP)-binding cassette (ABC) transport system to capture oligosaccharides digested before entering cells^26,27^. No strain has been identified to utilize all glycans; however, specific types of glycans are utilized. Metabolic specificity exists between different bifidobacteria and other gut microbiota in the range, species, and utilization strategies of available glycans, which induces interactions between bifidobacteria and other flora, including cross-feeding, to promote the gut adaptability of bifidobacteria^28^.

The utilization of HMOs by bifidobacteria is an example of occupying a specific trophic niche in the gut. *B. longum* and *B*. *bifidum* establish a cross-feeding relationship with other microbes (such as *Eubacterium*) in early life by sharing their HMO metabolites degraded by extracellular glycosidases, including fucose and short-chain fatty acids (SCFAs)^29,30^. This cross-feeding strategy can augment the accessibility of glycans to gut microbiota and mediate interspecific interactions through metabolite reuse, directly affecting host gut homeostasis and health^31,32^.

Understanding glycan utilization and cross-feeding mechanisms will help to facilitate the discovery of new genes, enzymes, and metabolites that are potentially involved in bifidobacteria-microbe associations and host health. Therefore, this review describes the glycan utilization properties of bifidobacteria and examines the glycan-based cross-feeding activities and mechanisms involved in bifidobacteria, as well as their impact on host health.

## Glycan preference and metabolic pathway of bifidobacteria

**Fig. 1:**
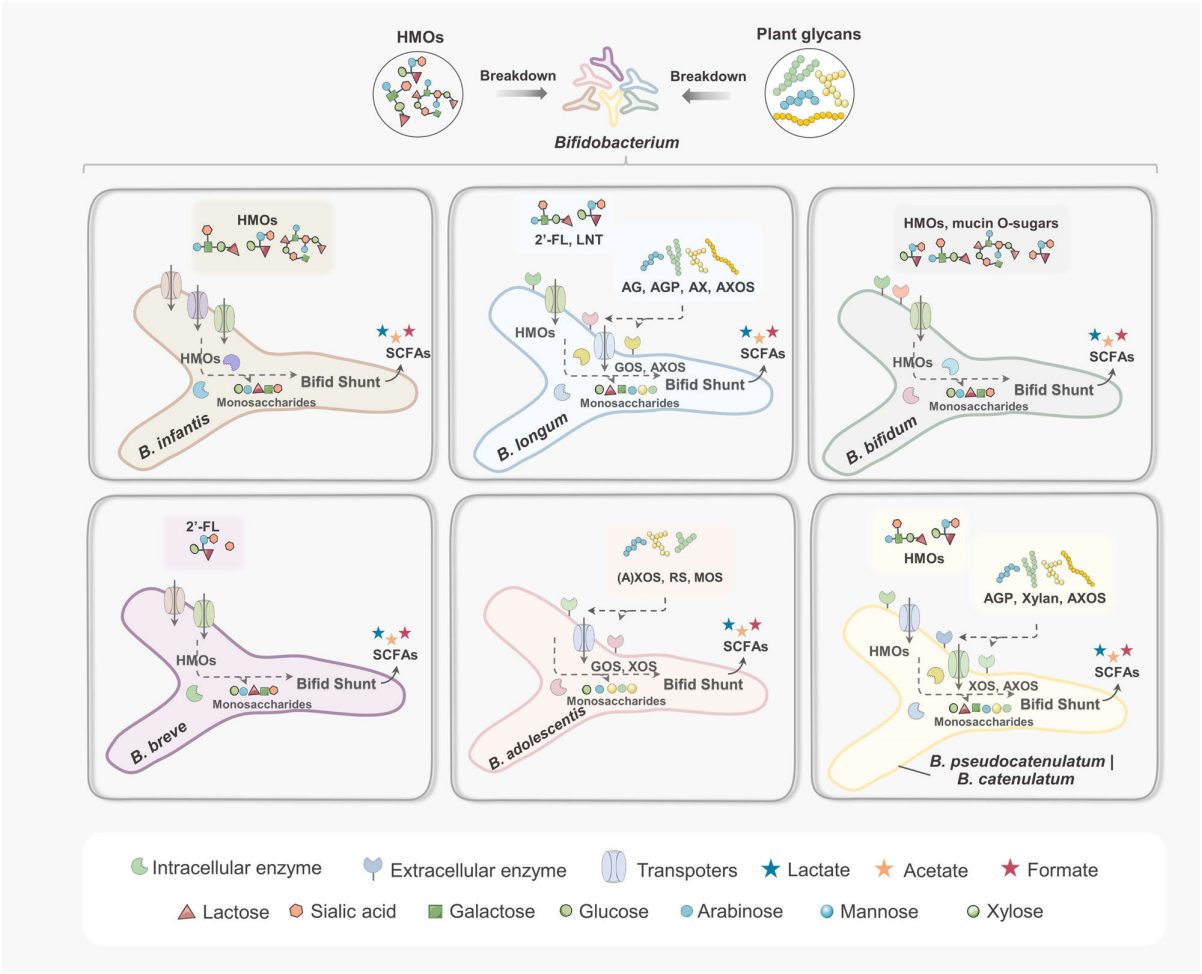
Major glycan utilization strategies of bifidobacteria. Bifidobacteria complete the degradation by cleaving the glycan by extracellular enzymes or internalizing the glycan directly. *B*. *infantis* can internalize most of the HMOs through the transporter system, and *B*. *bifidum* degrade the HMOs (including fucosylated glycans) by abundant extracellular enzymes for further internalization and uptake. *B*. *breve* utilize limited HMOs (mainly 2ʼ-FL). *B*. *longum* excel in the utilization of phytoglycans containing arabinose structures. *B*. *adolescentis* is an excellent utilizer of resistant starch. Both *B. pseudocatenulatum* and *B. catenulatum* can consume some HMOs and phytoglycans.

The colonization advantages of bifidobacteria depend on the availability, demand, and consumption rates of specific nutrient resources. The different carbohydrate metabolisms of bifidobacteria in different periods influence their selective adaptation to different gut environments. Due to differences in trophic sources, the intestinal environment of infants differs from that of adults, characterized by bifidobacteria including *B. longum* subsp. *infantis* (*B*. *infantis*), *B*. *breve*, and *B*. *bifidum*, which use HMOs as the primary carbon source; however, when introducing complex diets after weaning, the bifidobacteria *B. longum* subsp. *longum* (*B*. *longum*), *B*. *adolescentis*, *B. catenulatum*, and *B*. *pseudocatenulatum*, which have a greater capacity to degrade plant glycans, are more prevalent^6,33,34^. The breakdown of glycans by bifidobacteria depends on a significant number of GHs located extracellularly or within the cell wall. Bifidobacteria have evolved several homologous enzymes with overlapping, yet different, substrate specificities. Different species have different intestinal adaptations to cope with different glycan structures. Complex glycans are first degraded by extracellular GHs into monosaccharides or oligosaccharides, some of which are transported directly to the cytoplasm by transport proteins, while others are phosphorylated. These monosaccharides or oligosaccharides, and other simple carbohydrates, enter the bifid-shunt pathway (fructose-6-phosphate phosphoketolase central fermentative pathway) of bifidobacteria and are metabolized and used for ATP production^35^. The metabolic pathways of bifidobacteria are closely associated with the type and concentration of extracellular sugars in the gut. Bifidobacteria normally produce various metabolites, including acetate, ethanol, and formate, depending on the amount of sugar available; when extracellular sugars are abundant, acetate and lactate are generated^36^. Therefore, bifidobacteria adopt different strategies to colonize, and the capture, degradation, and metabolism of glycans by bifidobacteria can determine their trophic niches in the gut tract (Fig. 1 and Table 1).

**Table 1:** Major glycan utilization strategies of bifidobacteria

Bifidobacterial strains	Types of glycan	Strategies for glycan processing	Metabolites	References
***B. infantis***				
ATCC 15697	LNT and LNnT	Hexosaminidase hydrolyzes β 1-3 bonds in LNT and LNnT to release GlcNAc, which is then deacetylated by GlcNAc-6-P deacetylase (nagA) and deaminated by glucosamine-6-P isomerase (nagB).	GlcNAc, acetate, ethanol, formate, and lactate	^149^
Bi-26	2ʼ-FL	2ʼ-FL is transported by a special ABC transporter, and the genes encoding fucose peroxidase and ATP transporter are up-regulated during fermentation.	Fucose, acetate, lactate, 1,2-PD, and formate	^150,151^
EVC001	Human milk glycoproteins	Endo-β-N-acetylglucosaminidase releases N-glycans.	Lactate and acetate	^152^
***B. longum***				
105-A	Lactulose (contains Gal-β1, 4-Rha structure)	The SBP encoded by the BL105A 0502 gene internalizes lactulose. Gh42β-galactosidase is a candidate enzyme for Gal-β1, 4-RHA degradation.	Acetate and lactate	^153,154^
M12	2ʼ-FL and LNT	*B*. *longum* M12 contains the GH95 gene (α-1-2-L-fucosidase), which can grow on 2ʼ-FL and LNT as the sole carbon source, but lacks GH29 (α-1,3/4-fucosidase) and cannot utilize LNnT.	--	^46^
JCM7052	Gum arabic AGP	α-l-Rhap-(1 → 4)-β-d-GlcpA-(1 → 6)-β-d-Galp-(1 → 6)-d-Gal tetrasaccharide is produced by the cooperation of three extracellular enzymes including BlArafE, which is a new α-1-arabinofuranosidase (GH43/34) for splitting up the α1,4-Araf linkage.	Oligosaccharides (tet-rasaccharide S4)	^122^
JCM7052	AGP	3-O-α-D-galactosyl-α-L-arabinofuranosidase (GAfase) (GH39) has responsibility for the release of α-D-Galp-(1 → 3)-L-Ara and β-L-Arap-(1 → 3)-L-Ara.	L-arabinose, and galactose	^54^
JCM1217	Type II AG, and larch wood AG	β-1,6-galactobiose is produced by the combination of three enzymes including GH43_24 exo-β-1,3-galactanase (Bl1,3 Gal), GH30_5 exo-β-1,6-galactobiohydrolase (Bl1,6 Gal) and GH43_22 α-L-arabinofuranosidase (BlArafA).	β-1,6-galactobiose, and arabinofuranose	^16^
NCC2705	High-mannose N-glycan	After cleaving by an endo-β-N-acetylglucosaminidase (GH85), N-glycan is broken down by three GH38 α-mannosidases and a GH125 α-1,6-mannosidase.	Mannose, acetate formate, and ethanol	^56^
NCIMB 8809	Hydroxycinnamic acids (HCAs)	The CaeA esterase in an arabinoxylan/arabinan metabolism cluster can cleave several HCA-containing oligosaccharides.	---	^155^
***B. breve***				
UCC2003	Lacto-N-biose (LNB)	Three transcriptional regulators (LntR, NahR, and NagR1) are involved in regulating LN (n) T/LNB metabolism.	---	^156^
UCC2003	4-galactosyl-kojibiose and lactulosucrose	β-galactosidase and the specific gene clusters (Bbr_1551 to Bbr_1553) are used to degrade GOS and lactulose.	--	^157^
DSM 20091	GOS	GosDEC, GalCDE transporters, and extracellular GH53 enzymes are used to degrade GOS.	--	^158^
JCM1254, JCM7004, TMC3108, and TMC3115	2ʼ-FL, 3ʼ-FL, LNnT, and LNFP I	A combination of seven extracellular GH enzymes degrades HMOs, releasing degradants into the extracellular space.	LNB, lactose, galactose, and fucose	^66^
***B. bifidum***				
JCM1254	LNT	LNBase (LnbB) is specific for LNT degradation.	lacto-N-biose I and lactose	^45^
JCM 1254	Mucin O-glycans	Degradation of mucin O-glycans by GH 20 sulfoglycosidase (BbhII) and GlcNAc-6S-specific carbohydrate-binding module (CBM) 32.	N-acetylglucosamine-6-sulfate	^71^
***B. adolescentis***				
P2P3	High amylose corn starch	RSD1/2/3 and starch-binding modules (CBM25, CBM26 and CBM74) are used for RS degradation.	Maltooligosaccharides	^72^
DSMZ 20083	β-manno-oligosaccharide (MOS)	ABC and MFS transporters facilitate the uptake of linear MOS, while GH1 β-glucosidase and GH32 β-furanoglycosidase catalyze the cleavage of MOS.	Acetate, lactate, and formate	^77^
ATCC 15703	AXOS (DP 2–4 and mono-substituted)	GH43 α-L-arabinofuranosidase is responsible for degradation.	Lactate and acetate	^159^
***B. pseudocatenulatum***				
MP80	2ʼ-FL	A series of gene clusters containing GH29 and GH95 enzymes perform degradation of fucosylated HMOs.	1, 2-PD	^160^
JCM 1200	Sucrose (Suc) and N-acetyl sucrosamine (SucNAc)	Sucrose phosphorylase is responsible for Suc degradation, and β-fructofuranosidase is for SucNAc.	--	^85^
ED02	XOS and linear xylan	An extracellular GH10 endo-β-1.4 xylanase exhibits activity against both XOS and xylan.	XOS fractions of the various DP	^161^
YIT 4072 ^T^	Arabinoxylan hydrolysate (AXH)	Five GH43 enzymes and three transporters participate in the degradation of AXOS and XOS.	Arabinose and xylose	^162^
***B. catenulatum subspecies kashiwanohense***				
JCM 15439^T^	AX, xylan, and XOS	Extracellular xylanase can cleave AX into XOS and AXOS, which are subsequently further catabolized by intracellular arabino-franosidase and xylosidase into arabinose and xylose.	Arabinose and xylose	^87^
YIT 13060	2ʼ-FL, lacto-N-difucohexaose (LNDFH)	2ʼ-FL and LNDFH are translocated intracellularly and further degraded in cooperation with fucosidase, β-galactosidase, and Lacto-N-biosidases.	GLcNAC, fucose, galactose, and glucose	^87^

### *B. infantis* primarily internalizes HMOs

The ability of *B*. *infantis* to intracellularly utilize most HMOs provides a competitive advantage over other HMO-consuming species (such as *B*. *breve*), resulting in its dominance in the infant gut during breastfeeding^37^. The processing of 2ʼ-fucosyl lactose (2ʼ-FL) by *B*. *infantis* depends on the ABC transporters for intracellular digestion. *B*. *infantis* contains several intracellular enzymes including fucosidase, β-galactosidase, LNB phosphorylase, N-acetyl-β-hexosaminidase, and sialidase, to degrade fucosylated and sialylated HMOs^38^.

Transporter specificity is necessary for the effective uptake of HMOs. Sakanaka et al.^26^ have reported that the two FL transporters (FL1-BP and FL2-BP) of *B*. *infantis* can be used to absorb 2ʼ-FL and 3ʼ-FL, and the expression of FL transporters influences the abundance of *B*. *infantis*. Intracellular FL is utilized by α-fucosidase to produce fucose, which results in the secretion of 1,2-propanediol (1,2-PD), acetate, and lactate^39^. Non-fucose glycosylated neutral HMOs, including lactose-N-tetrasaccharide (LNT), lacto-N-neotetraose (LNnT), lactose-N-bisaccharide (LNB), and N-acetyl glucosamine, stimulate the growth of *B*. *infantis*^35,40^. Duar et al.^41^ determined that the utilization of LNT and LNnT by *B*. *infantis* EVC001 is closely associated with the Blon2175-2177 ABC transporter in its H5 cluster, which contains a complete gene repertoire of HMO utilization, while the H5-negative strains exhibit growth defects on the carbon source.

### *B. longum* can metabolize arabinoxylan

Colonization by *B. longum* dominates the entire lifespan of the host^42^. The abundance of *B. longum* is closely associated with food intake at different growth stages, while the gene pool for glycan acquisition and metabolism can be selectively altered due to changes in certain dietary components^43,44^. The colonization superiority of *B. longum* is manifested in its ability to grow on HMOs and metabolize complex arabinoxylan (AX) substrates.

Some *B. longum* metabolize specific HMOs, including LNT and 2ʼ-FL rather than sialylated HMOs, as well as mucin O-glycans, thereby occupying trophic niches in the gut during early life^45^. Sakanaka et al.^45^ identified an extracellular lacto-N-biosidase (LnbX) from *B. longum* JCM1217 that could consume LNT and release LNB and lactose via the GNB/LNB pathway. Díaz et al.^46^ found that *B. longum* M12 with α-1-2-L-fucosidase (GH95) could grow on 2ʼ-FL and LNT.

The transporters and GHs of *B*. *longum* are more likely to metabolize plant-derived glycans (dominated by various arabinose-substituted glycans)^47^. The enrichment of the gene cluster encoding L-arabinofura (-Araf) facilitates *B*. *longum* utilization of these glycans and reflects its ecological adaptability and competitiveness in the adult and aging gut environments^48^. The GH43 family, which includes α-L-arabinofuranosidase, β-xylosidase, arabinosidase, and xylanase, is substrate-specific and degrades insoluble fibers through cooperation. *B. longum* has an abundant GH43 gene cluster capable of releasing arabinoxylooligosaccharides (AXOS), arabinose and xylose from arabinogalactan (AG), arabinogalactan (AN), and AX^16,49,50^. Collectively, the ability of *B*. *longum* to degrade AX and AXOS is strain-specific and influenced by different priorities for utilizing different monosaccharide compositions and structures (such as degree of polymerization (DP), linkage type, and side-chain space structure)^51^. For example, *B*. *longum* JCM 1217 may prefer natural and partially degraded AX components with higher DP, side chain content, and arabinosyl-monosubstituted structure, possibly due to differences in the transport system specificity and membrane localization of the enzymes^14^. AG and arabinogalactan protein (AGP) are complex fiber components in plant cell walls that are consumed only by a few sub-species of *B*. *longum*; most bifidobacterial species cannot absorb full AGP^52^. Fujita et al.^16^ reported a type II AG-degrading enzyme in *B*. *longum* JCM1217 that can grow on larch AGP but cannot act on gum arabic AGP with a more complex structure. The high molecular weight arabinose side chain in the highly modified AGP causes steric hindrance, resulting in limited cleavage of β-1,3/1,6 bonds in the galactose skeleton by β-galactosidase and arabinoglycosidase and insufficient galactose and galactooligosaccharides (GOS) release^53^. Sasaki et al.^54^ identified a novel arabinofuranosidase (named GAfase) that can cleave the l-Ara structure of gum arabic AGP to remove steric hindrance, a key factor in the growth of *B*. *longum* JCM7052 in gum arabic AGP. These relatively limited degradations suggest that *B*. *longum* must cooperate with other bacteria to completely consume the AGP and AG complex. Moreover, *B*. *longum* selectively utilizes other N-glycans for growth. Specifically, the α-mannosidases, N-acetyl glucosaminidase, and α-glucosidase of *B*. *longum* NCC2705 release mannose and N-acetyl glucosamine (GlcNAc) from mannans through cooperation^55,56^.

### *B. breve* has limited growth on complex HMOs

HMO metabolism in *B*. *breve* is highly similar to *B. infantis*, which absorbs intact oligosaccharides through the ABC transport system and degrades them in cells. However, the complete gene pool required to metabolize complex HMOs, such as fucosyllactose and sialyl-lactose, and the utilization of HMO components by *B*. *breve* are limited to N-glycans, including LNT, LNnT, and N-disaccharide (LNB)^57,58^. The genome of *B*. *breve* mostly contains genes involved in the breakdown of lactose, fucose, sialic acid, and amino sulfate^59–61^. Specifically, the external sialidase (GH33) in *B*. *breve* can help to release sialic acid from mucin and grow on free sialic acid in the gut environment^29^. The N-acetyl glucosamine 6-phosphate deacetylase of *B*. *breve* UCC2003 can efficiently break down GlcNAc-6-sulfate residues in O-glycans^61^. Bottacini et al.^62^ analyzed the genomes of 20 *B*. *breve* strains and determined that α-glucosidases (GH13 and GH31), β-glucosidase (GH1 and GH3), and β-galactosidases (GH2 and GH42) were involved in the metabolism of α-glucans (such as maltose), cellobiose, and galactose, respectively. Notably, most *B*. *breve* strains cannot utilize fucosylated HMOs (2ʼ-FL or 3ʼ-FL), but utilize fucose produced by other bacteria^63^.

### *B*. *bifidum* extracellularly degrade HMOs and mucin O-sugars

Compared with other bifidobacterial species, *B*. *bifidum* has a complete set of extracellular GHs, including α-sialidase, α-fucosidase, N-Acetyl-β-hexosaminidase, β-galactosidase, and LNBase, to assimilate complex HMOs and leave degradation products, such as lactose, fucose, and sialic acid, outside the cell^45,64–66^. Nishiyama et al.^67^ identified a sialidase (SiaBb2) involved in the degradation of HMOs and mucin and may promote the adhesion and colonization of *B*. *bifidum* on the surface of the intestinal mucosa. Additionally, *B*. *bifidum* lacks specific enzymes to degrade N-glycans, however, has a substrate preference for O-glycans, which avoids competition with other bifidobacterial species in the guts of breast-fed infants. *B*. *bifidum* encodes N-acetyl galactosidase (GH101) and Lacto-N-biosidase (GH136), which degrade mucin O-glycosidic bonds, and has abundant carbohydrate-binding modules (CBMs) that promote the proximity of GHs to substrates^68,69^. Takada et al.^70^ found that the β-N-acetylglucosaminidases of *B. bifidum* can specifically degrade β-GlcNAc linkages of mucin core structures, while Katoh et al.^71^ reported that *B. bifidum* could release GlcNAc-6S from sulfated O-glycans using sulfoglycosidase and may affect the metabolism of other bacteria.

### *B*. *adolescentis* prefers to degrade starch

The competitive advantage of *B*. *adolescentis* is mainly reflected in its utilization of plant glycans, including RS, mannooligosaccharides (MOS), and inulin. *B*. *adolescentis* contains α-amylases, glycogen debranching enzyme, pullulanase, and a specific starch-binding module, which facilitate the complete degradation of RS^72,73^. Enzymes encoding other glycans, including galactosidase, mannosidase, β-xylosidase, and arabinofuranosidase, similarly reflect the preference of *B*. *adolescentis* in degrading dietary glycans^74^. Moreover, Mulualem et al.^75^ identified an α-galactosidase (BgaC) from *B*. *adolescentis* ATCC15703 that produces GOS from lactose through transglycosylation. Notably, *B*. *adolescentis* consumes oligosaccharides and is more likely to grow on MOS (DP ≤ 4) and mannose, using β-glucosidase (GH1) and β-fructofuranosidase (GH32), than complex β-mannan^76,77^. Salas-Veizaga et al.^78^ reported that *B*. *adolescentis* metabolizes glucuronosylated-XOS (GXOs) and XOs (DP 2-6) to produce SCFAs. *B*. *adolescentis* prefers (A)XOS to arabinose or xylose, and encodes a gene for endo-1,4-β-xylanase capable of growing on wheat AX^9,14,79^.

### *B*. *pseudocatenulatum* and *B. catenulatum* consume host and plant glycans

*B*. *pseudocatenulatum* and *B. catenulatum* are found in the guts of infants and adults. Their CAZyme genes differ in hosts of different ages^6,80^, suggesting that both species have a relatively well-developed degradation system for both host and plant sugars.

The α-fucosidases GH29 and GH95 induced *B*. *pseudocatenulatum* MP80 to exclusively utilize lower-molecular-weight fucosylated HMOs, such as lactodifucotetraose^81^. *B*. *pseudocatenulatum* has an endo-1,4-β-xylanase (GH10) that can thrive on long-chain xylan-derived polysaccharides (XOS with polymerization degrees 2–4), which may become a critical feature of *B*. *pseudocatenulatum*, consistent with the finding that bifidobacteria have a limited ability to degrade xylan^82^. Additionally, endo-1,4-β-xylanase cuts the main chain of xylan to produce XOS and xylose. While some metabolites are absorbed by the intracellular utilization system, others are released into the extracellular environment for use by secondary consumers^82^. Specifically, a β-L-arabinopyranosidase (AAfase) is characterized in the genome of *B*. *pseudocatenulatum* MCC10289 for the assimilation of AGP side chains and L-arabinose^83^. Hosaka et al.^84^ determined that *B*. *pseudocatenulatum* JCM 1200 expresses sucrose phosphorylase and β-fructofuranosidase to hydrolyze sucrose and analog disaccharide N-acetyl sucrosamine (SucNAc)^85^.

Similarly, the genome of *B. catenulatum* contains genes for GHs involved in HMOs and xylan, starch, and their derived oligosaccharides, which can adapt to changes in the host diet^86^. HMOs, including 2ʼ-FL and lacto-N-difucohexaose, are translocated intracellularly by *B. catenulatum* and degraded into GLcNAC and monosaccharides in cooperation with fucosidase, β-galactosidase, and Lacto-N-biosidases^87^. The extracellular xylanase of *B. catenulatum* can cleave AX into XOS and AXOS, which are subsequently catabolized by intracellular arabino-franosidase and xylosidase into arabinose and xylose^87^.

## Glycan utilization capacity of bifidobacteria shapes cross-feeding interactions with other species

**Fig. 2:**
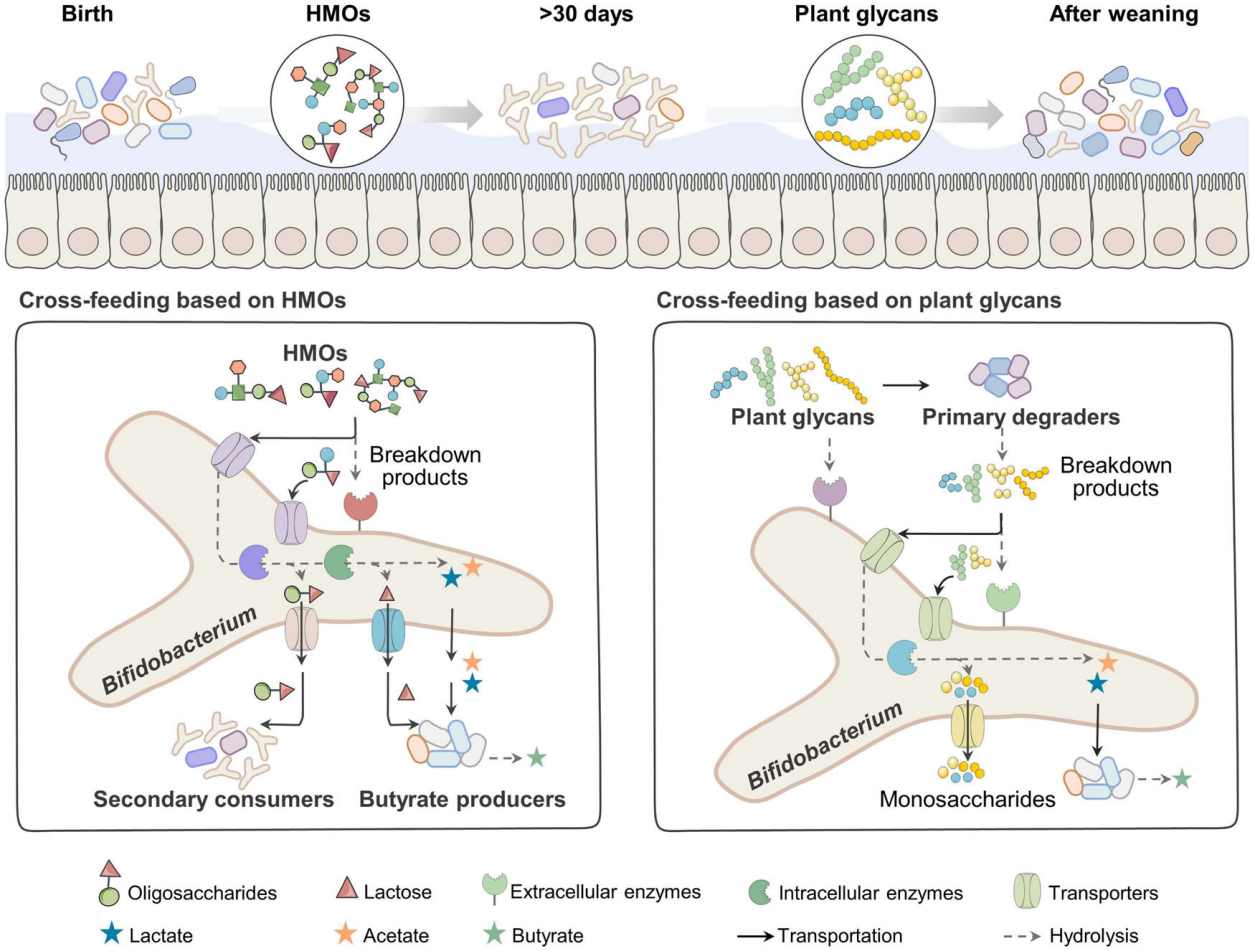
Cross-feeding strategy of bifidobacteria throughout the life span. Cross-feeding of bifidobacteria exists throughout the life cycle. The major categories are HMO-based and phytoglycan-based cross-feeding. Specifically, when co-cultured on HMOs, bifidobacteria are more likely to cross-feed HMO degradation products to other non-HMO degradation-dominant bacteria and butyrate producers. When cross-fed on phytoglycans, bifidobacteria are more likely to thrive by relying on the extracellular degradation products of other dominant degrading bacteria.

The gut microbiota is mostly auxotrophic, and the assimilation of glycans by different species may be similar (metabolic redundancy). Therefore, there remains a need to compete with other species or obtain nutrition from the gut environment^88,89^. Relying on other microorganisms that release metabolites into the environment, competing species can coexist in equilibrium; these cost-free metabolites promote microbial interactions^90^. Cross-feeding exists between species with different metabolisms, specifically those that utilize particular complex compounds to liberate metabolic by-products that are further assimilated by others that cannot grow on those compounds alone^28,90,91^. Bifidobacteria rely on their enzymatic breakdown and oligosaccharide transport systems to consume glycans from the host, resulting in stable colonization of the gut tract and facilitating cross-feeding relationships within bifidobacterial species or with other bacteria, such as *Bacteroides* and butyrate producers^92^ (Fig. 2 and Table 2).

**Table 2:** Cross-feeding strategies of bifidobacteria based on glycan utilization

Bifidobacterial strains	Cross-feeders	Types of glycan	Secondary and final metabolites	Strategies for cross-feeding	References
**Cross-feeding based on HMO and Mucins**					
*B*. *infantis* ATCC15697	*A. caccae* L1-92	HMOs	Lactose, lactate, and acetate	*B*. *infantis* releases metabolites from HMOs to help *A. caccae* produce butyrate.	^93^
*B*. *longum* BSM11-5, *B*. *breve* DSM 20213, and *B*. *infantis* DSM 20088	*E. hallii* 3353	Fucosyllactose and L-fucose	Glucose, lactate, acetate, and 1, 2-PD	L-fucose is metabolized to produce lactate, 1, 2-PD, and acetate, which are further converted to butyrate, formate, and propionate by *E. hallii*.	^30^
*B*. *longum* LH206	*B. pseudocatenulatum* LH657, LH659	LNnT	2ʼ-FL and galactose	The products of LNnT are degraded by *B. longum* and are cross-fed to *B. pseudocatenulatum*, and the product of 2ʼ-FL is metabolized by *B. pseudocatenulatum*, to support the growth of *B. longum*.	^29^
*B*. *bifidum* ATCC 15696	*B*. *breve* 24b	2ʼ-O-Fucosyl-Lactose	Lactose and fucose	Lactose and fucose are cross-fed to *B*. *breve* 24b for 1, 2-PD production.	^98^
*B*. *bifidum* CCX 19041 and *B*. *infantis* CCX 19042	*B*. *breve* CCX 19061	Sialylated immunoglobulin G	N-acetylneuraminic acid (Neu5Ac) and galactose	Neu5Ac and galactose degraded by *B*. *bifidum* CCX 19041 and *B*. *infantis* CCX 19042 are cross-fed to *B*. *breve* CCX 19061.	^100^
*B*. *bifidum* PRL2010	*B*. *breve* UCC2003	3ʼ-SL	Sialic acid and lactose	*B*. *breve* uses the extracellular degradation products of *B*. *bifidum*.	^163^
*B*. *bifidum* ATCC 15696	*B*. *breve* JCM 7019	6ʼ-SL	Sialic acid and lactose	*B*. *breve* JCM 7019 consumes the hydrolysates by extracellular sialidase of *B*. *bifidum* ATCC 15696.	^99^
*B*. *bifidum* BSM28-1	*B*. *breve* BRS 26-2, *B*. *infantis* DSM 20088, and *E. hallii* DSM 3353	Mucins	Lactose, formate, and acetate	*B*. *bifidum* BSM28-1 provides lactose to the other three strains, and *E. hallii* consumes secondary metabolites for butyrate and propionate production.	^94^
*B*. *breve* UCC2003	*Lm. reuteri* ATCC PTA 6475	Fucose	1, 2-PD	The pduCDE operon of *L. reuteri* is responsible for utilizing 1, 2-PD produced by *B*. *breve* UCC2003.	^102^
**Cross-feeding based on plant glycans**					
*B*. *longum* LMG 11047	*L. paracasei* 8700:2 and *Anaerotipes caccae* DSM 14662 ^T^, or *E. hallii* DSM 17630	ITF	Oligofructose, lactate, and acetate	*B*. *longum* LMG 11047 grows on short-chain inulin produced by *Lactobacillus* and releases acetate and lactate which are further converted to butyrate and gases.	^118^
*B*. *longum* NCC2705	*E. rectale* ATCC 33656	AXOS	Acetate, arabinose, and xylose	Both bacteria could degrade XOS. *E. rectale* ATCC 33656 uses acetate produced by *B*. *longum* to produce butyrate and xylose; the latter is consumed by *B*. *longum*.	^108^
*B*. *longum* NCC 2705	*B. caccae* ATCC 43185	Larch wood AG	Carbohydrate fragments of AG, lactate	*B*. *longum* NCC 2705 depends on the products of AG degradation by *B. caccae* ATCC 43185, while *Bacteroides* may utilize lactate produced by *B*. *longum*.	^53^
*B*. *longum* BB536	*A. caccae* DSM 14662 and *Roseburia intellalis* DSM 14610	FOS	Acetate and fructose	*A. caccae* DSM 14662 grows on fructose released by *B*. *longum* BB536, while *Roseburia intellalis* DSM 14610 consumes acetate.	^164^
*B*. *adolescentis* L2-32, and *B*. *adolescentis* DSM 20083	*E. hallii* L2-7, and *R. homini* A2-183	Starch or FOS	Lactate, acetate, and oligosaccharides	Lactate and acetate could be cross-fed to *E. hallii*, and oligosaccharides could be used as growth substrates for *R. hominis*.	^104^
*B*. *adolescentis* ATCC 15703	*R. hominis* A2-183	Linear or galactose-substituted β-mannan-oligosaccharides	Acetate	Acetate helps *R. hominis* A2-183 produce butyrate and grow on mannan-oligosaccharides.	^109^
*B*. *adolescentis* DSMZ 20083	*B. ovatus* DSMZ 1896	Galactomannans	β-mannooligosaccharides	*B*. *adolescentis* DSMZ 20083 relies on short β-mannooligosaccharides (DP3).	^115^
*B*. *adolescentis* L2-32	*F. prausnitzii* S3/L3	FOS	Acetate and FOS residues	*B*. *adolescentis* cross-feeds acetate to *F. prausnitzii* and grows better due to FOS residues released by *F. prausnitzii*.	^165^
*B*. *adolescentis* LMG10734	*F. prausnitzii* DSM 17677^(T)^	ITF	Acetate	Acetate is used for butyrate production.	^110^
*B. pseudolongum* ST6	*B. animaiis* subsp*. lactis* RG1	Hi-Maize starch	Maltose	Maltose is released by *B*. *pseudolongum* and cross-fed to *B*. *animaiis*.	^111^
*B*. *breve* UCC2003	*Bacteroides cellulosilyticus* DSM 14838 (*Baccell*)	Larch wood AG	β-1,3-GOS	*B*. *breve* UCC2003 uses the short β-1,3-GOS released by *Baccell*, eventually producing succinic acid and acetate.	^166^
*B*. *bifidum* PRL2010	*B*. *breve* 12 L, *B*. *adolescentis* 22 L, or *Bacteroides thermophilum* JCM1207	RS2-resistant starch or xylan	Glucose and or maltose	*B*. *bifidum* PRL2010 may utilize glucose and or maltose released by other bifidobacterial strains, and the genes involved in glycolysis in these species were upregulated.	^112^
*B*. *animalis* subsp. *lactis* DSM-10140	*B. ovatus* DSM-1896 or *Bacteroides xylanisolvens* DSM-18836	Beechwood and corncob xylans	XOS	*B*. *animalis* subsp. *lactis* grows on XOS produced by *Bacteroides*, releasing lactate and acetate.	^19^

### HMO-based cross-feeding of bifidobacteria allows for its dominance in the guts of infants

#### Cross-feeding of bifidobacteria with butyrate producers based on HMOs

*B*. *infantis* metabolizes HMOs to produce oligosaccharides and metabolites (such as 1, 2-PD and acetate) that play important roles in the gut tract of breastfed infants by cross-feeding with other bacteria such as *Eubacterium hallii* and *Anaerostipes caccae*^30,93,94^.

Cheng et al.^95^ found that *B*. *infantis* utilizes 6ʼ-sialyllactose (6ʼ-SL) for acetate production and is cross-fed to *Faecalibacterium prausnitzii* for butyrate conversion, which causes *B*. *infantis* to proliferate as a result. This may be associated with the fact that *F. prausnitzii* secretes extracellular sialidase to promote the expression of sialidase in *B*. *infantis*, thereby increasing acetic acid production, and that the occurrence of this cross-feeding activity depends on the molecular structure of HMOs^95,96^.

#### Cooperative degradation of HMO between bifidobacterial species

When extracellularly degrading HMOs, the products produced by bifidobacteria (including *B. bifidum* and *B. longum*) are partially released into the public environment, inducing cross-feeding with other bifidobacterial species with weaker HMO-utilizing capacity.

For example, the products of LNnT degradation by *B. longum* could be cross-fed to *B*. *pseudocatenulatum*, and the oligosaccharides from 2ʼ-FL degradation by *B*. *pseudocatenulatum* support the growth of *B. longum*^29^. *B*. *bifidum* is not the dominant species in the intestinal tract of infants; however, as an extracellular degrader of HMOs, it cross-feeds with other species by releasing HMO derivatives, thus affecting the composition of the gut microbiota^97^. HMO metabolism between *B*. *bifidum* and *B*. *breve* is complementary, which also contributes to cross-feeding between the two strains^61^. *B*. *bifidum* uses fucosidase and FL transporters to release lactose and fucose extracellularly, which cross-feeds to *B*. *breve*. This indicates that *B*. *bifidum* is easy to cross-feed with other fucose consumers who lack extracellular fucosidase. However, *B*. *bifidum* prefers to grow on lactose rather than fucose, suggesting altruism as its primary function^98^. In addition to cooperation, *B*. *breve* can compete with *B*. *bifidum* for lactose; the key to coexistence is that *B*. *bifidum* releases more lactose by upregulating the gene expression of the enzymes and transporters involved^98^. A similar phenomenon was observed in the co-culture of mucin and sialylated glycan^59,99^. Chen et al.^100^ demonstrated that *B*. *bifidum* released sialic acid and lactose from sialylated glycans, which supported the growth of *B*. *infantis* and *B*. *breve* as secondary metabolites of cross-feeding. *B*. *bifidum* does not assimilate fucose, galactose, NeuAc, and GlcNAc-6S; therefore, the degradation of mucin O-glycan may be accomplished in cooperation with other species, such as *B*. *breve*, that utilizes these mucin-derived carbohydrate fragments^66,101^.

#### HMO-based cross-feeding of Bifidobacteria with other gut bacteria

Cross-feeding relationships established between bifidobacteria and other gut bacteria can help to refine the network of interactions within the gut microbiota during early life. Cheng et al.^102^ demonstrated that the metabolite 1,2-PD produced by *B*. *breve* through fermenting fucose could promote the colonization of *Limosilactobacillus reuteri* in the gut of gnotobiotic mice. Nogacka et al.^103^ investigated the 2-ʼFL-based metabolic interactions between *B. bifidum* and *Lactobacillus gasseri* and found that *B. bifidum* IPLA20048 promoted the proliferation of *L. gasseri* IPLA20136 by cross-feeding the extracellular degradation products (galactose, fucose, and lactose) and that genes encoding α-fucosidase involved in carbohydrate transport are upregulated in *B. bifidum* thereby increasing carbohydrate production.

The gut microbiota of breastfed infants undergoes significant changes in response to diet before and after weaning, thereby facilitating more complex microbial interactions and inducing gradual stabilization and maturation of the structure and composition of the gut microbiota.

### Phytoglycan-based cross-feeding of bifidobacteria after weaning enables its persistence in the gut

#### Cross-feeding of bifidobacteria with butyrate producers based on plant glycans

Acetic and lactic acids are the end products of oligosaccharides metabolism by bifidobacteria and are important substrates of cross-feeding between bifidobacteria and butyric acid bacteria^104–106^. When grown on AXOS, except for the conversion of acetic acid to butyric acid, butyrate producers consume AXOS to produce arabinose and xylose, which can be consumed as substrates by *B*. *longum*^107,108^. Bhattacharya et al.^109^ investigated the cross-feeding between *B*. *adolescentis* and *Roseburia* based on galactose-substituted β-mannan-oligosaccharides.

Moens et al.^110^ confirmed that bifidobacteria can easily cross-feed on FOS and inulin-type fructan to produce the bifidogenic and butyrogenic effects and that the conversion degree of acetic acid to butyric acid was closely associated with the differences in the glycan-degrading ability of bifidobacterial species. Specifically, when co-cultured with *F. prausnitzii*, *B. breve* can only utilize fructose, cannot consume FOS and ITF, and relies on the monosaccharides produced by *F. prausnitzii* to degrade fructan for growth. *B. adolescentis* prefers short-chain FOS over ITF, allowing it to cross-feed acetate to *F. prausnitzii* and utilize its FOS for continued growth. In contrast, *B. longum* or *B. angulatum* competes with *F. prausnitzii* on ITF, resulting in the lowest efficiency of butyric acid synthesis in the co-culture system^110^.

#### Phytoglycan-based cross-feeding between bifidobacterial species expands carbon source availability

When co-cultured, bifidobacterial species that cannot degrade plant glycans rely on other species that can degrade. For example, Centanni et al.^111^ reported that when grown on Hi-Maize, *B. pseudolongum* extracellularly produced type 1 pullulanase and alpha-amylase for the release of glucose, maltose, and maltotriose, which are cross-fed to *B*. *animalis*. More importantly, bifidobacterial species with similar nutrient metabolism can expand their trophic niches by cross-feeding the same substrates rather than competing. For example, *B. bifidum* PRL2010 lacks a degradation system that utilizes plant glycans, such as starch and xylan; however, when co-cultured with *B. adolescentis* 22 L, *B. brev*e 12 L, and *B. thermophilum* JCM1207, it can grow on simple carbohydrates released by other bifidobacteria and promote its sugar utilization gene expression^112^. When co-cultivated with other bifidobacteria, *B*. *adolescentis* exhibits the most significant alterations in genes involved in xylose metabolism compared with other species, reflecting the enhanced genetic adaptability of these strains, which may help hosts to expand the utilization potential of carbohydrates during specific dietary changes^113^.

#### Bifidobacteria cooperates with other gut bacteria to degrade complex plant glycans

Cross-feeding of breakdown products of complex glycans among the gut microbiota depends on the complexity of the target glycan. Due to the lack of a complete degradation and transport system, bifidobacteria have a limited capacity for recalcitrant plant glycans, including xylan, long-chain inulin, and mannan; therefore, they rely on other bacteria to extracellularly release soluble oligosaccharides as cross-feeding substrates, such as AXOS, XOS, FOS, and mannose^76,114^. Some members of the genera *Bacteroides* and *Lactobacillus* are dominant glycan-degrading bacteria that can cross-feed with *Bifidobacterium*, including *B. longum*, *B. animalis*, and *B*. *adolescentis*^19,115^. When grown on simple xylans, such as glucuronoxylan and wheat arabinoxylan, *B*. *adolescentis* ATCC 15703 relies on AXOS produced by *Bacteroides ovatus* ATCC 8483 for growth in the absence of a degradation system containing xylanase (GH98)^116^. The cross-feeding of fructans is closely associated with the substituents and length of the sugar chain, and complex fructans (i.e., those containing sucrose units) are degraded by primary degraders to produce short-chain compounds that are utilized by secondary consumers^117^. *B*. *longum* tends to use short-chain inulin, leading to cooperation with *Lactobacillus paracasei* to completely degrade long-chain inulin and produce fructose, lactate, and acetate^118^.

*B*. *longum* has a cross-feeding relationship with *Bacteroides* based on xlyan, type II AG, and AGP. Wang et al.^53^ reported that when co-cultured with *Bacteroides caccae* ATCC 43185, *B*. *longum* NCC 2705 thrived on the carbohydrate fragments of larch wood AG degraded by *B. caccae*. Vega-Sagardía et al.^119^ reported that *B. ovatus* HM222 degraded xylan and promoted the growth of *B. longum* PT4 by releasing XOS and extracellular enzymes and that the secretion of α-L-arabinoglycosidase, L-arabinose isomerase, and xylulose isomerase increased in *B. longum* PT4. However, due to the poor growth of *Bacteroides* at pH <5.5, with the production of acetate and lactate by *B*. *longum* fermentation, the substrate conversion (from succinic acid to propionate) efficiency of *Bacteroides* decreases, resulting in succinic acid accumulation^53^. Therefore, cross-feeding with *Bacteroides* is pH-limited, and the combined bacteria used to degrade complex AG or xylan require the participation of other species, such as *Prevotella* and *Roseburia intestinalis*^120,121^. Notably, the cross-feeding relationship between *B*. *longum* and *Bacteroides* is mutual, and the secondary products of plant glycan metabolism from *B*. *longum* can be cross-fed to some *Bacteroides* with a poor glycan metabolic system. For example, AGP-related oligosaccharides degraded by *B*. *longum* can be cross-fed with *Bacteroides vulgatus*, which promotes xylosidase expression in the latter strain^122^. Sonnenburg et al.^123^ determined that *B*. *longum* promoted the functions of mannosidase and xylosidase in *Bacteroides thetaiotaomicron* when co-cultivated on plant glycans. Given the limited data, whether the cross-feeding interactions involving bifidobacteria contribute positively to the balance of carbohydrate utilization within the entire intestinal community remains to be determined.

## Availability of glycans and cross-feeding activities shape the critical position of *Bifidobacterium* during life span

**Fig. 3:**
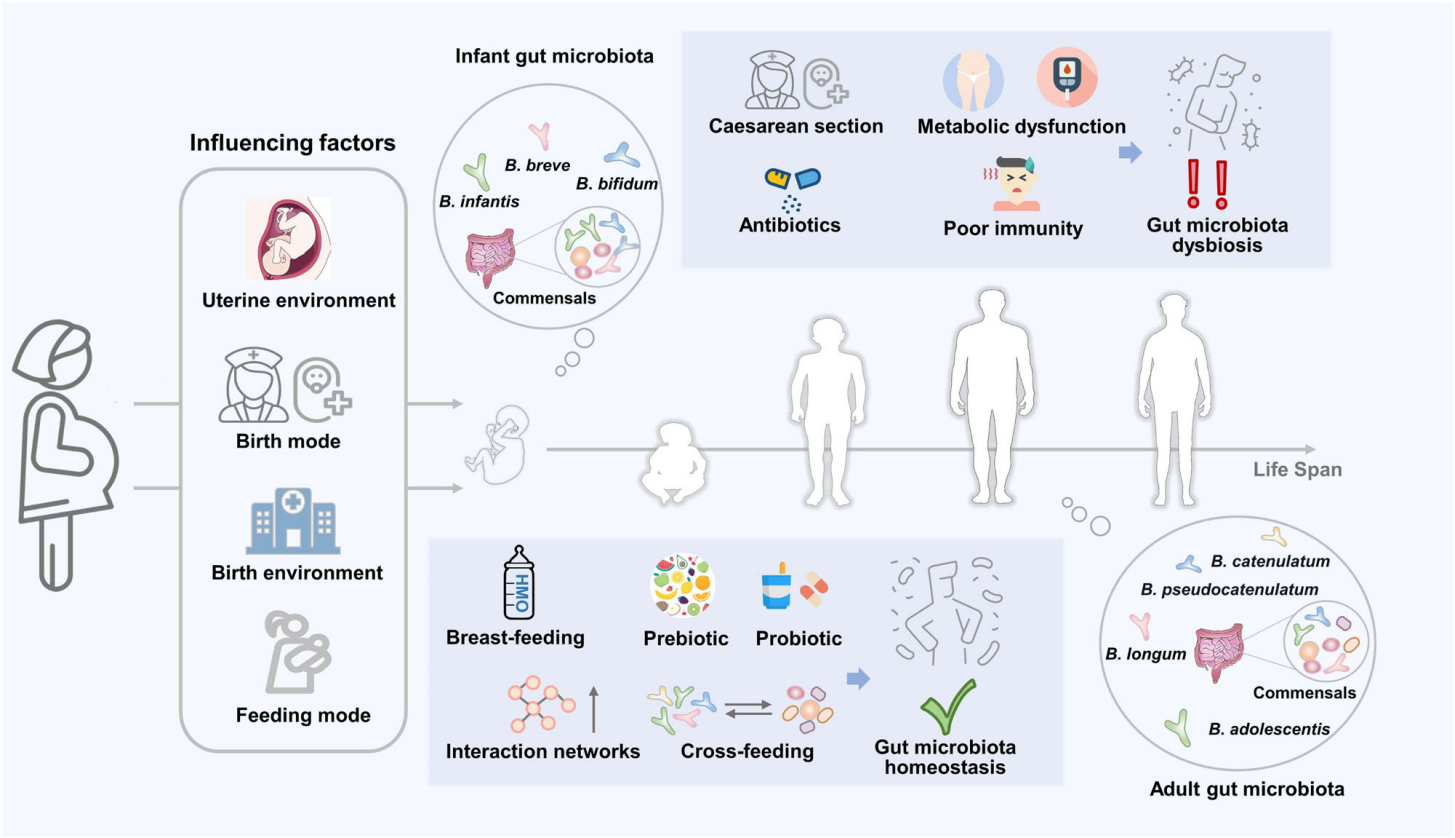
Benefits of cross-feeding of bifidobacteria throughout life span. The bifidobacteria of the host are influenced by several factors from birth. Breastfeeding and the use of probiotics and prebiotics contribute to the establishment of a network of interactions of the gut microbiota, including cross-feeding. The cross-feeding relationship can facilitate the formulation of multi-strain synbiotics, which are effective in improving intestinal homeostasis and mitigating related disorders including dysbiosis, metabolic syndrome, and immunodeficiency, due to cesarean section and antibiotic use.

The cross-feeding activities produced by bifidobacteria underscore their key ecological role in acquiring or providing substrates from or for other bacteria. This dependent interaction can also enhance the metabolic function of the participants, thus expanding their original trophic niche. The supplementation with bifidobacteria-containing multistrain synbiotics to construct a network of interactions between different members of the gut can help the gut microbiota to mature and stabilize during early life and increase the secretion of beneficial metabolites (such as butyrate), ultimately preventing chronic diseases associated with an imbalanced gut microbiota^44,124^. This synergistic combination of strains synergistically regulates gut microbiota and health homeostasis and is less affected by endogenous microorganisms than a single strain^125,126^. However, current research on the regulation of health by multi-strain synbiotics is only at the phenomenal description level, and clear explanations of the underlying reasons for the combination, the basis of its formulation, and the mechanism of its effectiveness remain scarce. Therefore, the possibility and necessity of such multi-strain synbiotics should be explored based on the preference of probiotics for glycans and the cross-feeding their relationships^127,128^. Gut microbiota is often affected by age, dietary habits, and antibiotics, necessitating tailored nutrition based on the needs of different hosts using appropriate and individualized strategies to regulate flora homeostasis^34,129^ (Fig. 3).

### Beneficial effects of cross-feeding involved in bifidobacteria during early life

#### Ameliorating the adverse consequences of microbial colonization caused by cesarean section and antibiotic use

Mode of birth (cesarean section) and antibiotic treatment negatively impact gut microbiota during infancy, as evidenced by the accumulation of pathogenic bacteria, decrease in *Bifidobacterium* and *Bacteroides*, and increase the risk of metabolic, inflammatory, and immune disorders in infants^127,130,131^. Supplementation with *Bifidobacterium* strains with extensive HMO metabolism (such as *B. infantis*), probiotics, and prebiotics that can stimulate the growth of *Bifidobacterium* have been used to reverse gut microbial dysbiosis induced by childbirth^132,133^.

*B. breve* has been reported to grow well on 2ʼ-FL by coexisting with other species encoding an extracellular fucosidase (GH95). Lou et al.^132^ found that supplementation with *R. gnavus* can promote the proliferation of *B. breve*, which utilizes lactose released by the former from the 2ʼ-FL, and contribute to the shift of the preterm microbiome to a bifidobacteria-rich community. Korpela et al.^127^ developed a mixture of *Lactobacillaceae rhamnoides*, *B*. *breve*, and *Propionibacterium freundenreichii* combined with FOS, which promoted the colonization of *Bifidobacterium* and reduced the abundance of Enterococcaceae, Clostridiaceae, and Veillonellaceae, ultimately correcting adverse changes in the gut microbiome due to cesarean section and antibiotic induction. A metaproteome analysis revealed that the enzymes used for HMO degradation in *Bifidobacterium* (including β-galactosidase and β-galactosyl N-acetyl hexosaminephosphorylase) were significantly expressed and *Lactobacillus rhamnosus* and might benefit from the monosaccharides released from HMO degradation^127^. These studies suggest that the administration of probiotics and/or prebiotics with gut-resident species (especially *Bifidobacterium*) as allies is effective in restoring gut microbiota disorders in infants; such multi-strain and prebiotic combinations with synergistic effects can be used as supplements in infant formulas.

#### Promoting a dominant position of bifidobacteria and perfecting intestinal community assembly

HMO-based metabolic interactions can shape the composition of infant gut microbiota. *Bifidobacterium* (especially *B. infantis*) is prioritized for gut colonization of breastfed infants, as determined by their superior ability to assimilate HMOs^21,134^. Bifidobacteria exert a priority effect based on HMO metabolism shortly after birth and consume most of the carbon sources in the gut, which prevents the colonization of later species, stimulates the establishment of infant intestinal homeostasis, and inhibits the growth of pathogens (such as *Clostridium difficile* and *Salmonella*)^135^. Intestinal colonization of *B*. *breve* is an example of priority effects. Considering the limited utilization ability of *B*. *breve* toward HMO, the formation of its dominant position is not simply related to the phenotype of HMO consumption but depends on cross-feeding with other bifidobacteria^61,66^.

The cross-feeding activity involved in bifidobacteria plays a key role in the assembly of gut microbiota. Chang et al.^136^ found that *B. infantis* Bg2D9 promoted the colonization of *Prevotella copri* Bg131 in the gut of malnourished mice and facilitated the release of arabinose from diets containing arabinoglycans, which contributed to the proliferation of other species in the gut, including *B. catenulatum* and *Blautia obeum*, by cross-feeding. This could be used as a dietary intervention in severely acutely malnourished children.

In summary, the gut microbiota is highly malleable after birth and it is possible to promote gut homeostasis by introducing diets with appropriate synbiotics. Determining the processes and outcomes of these interactions can be used for dietary interventions to achieve personalized nutrition^137^.

### Beneficial effects of cross-feeding involved in bifidobacteria during adulthood

#### Reestablishing gut microbiota structure and gut homeostasis

The prevalence of endogenous bifidobacteria is lower in adulthood than early life; however, due to the increase in dietary diversity, cross-feeding activities involved in bifidobacteria facilitate more interactions with the gut microbiota and host^34^. Dietary supplementation with multi-strain synbiotics helps to promote the proliferation of bifidobacteria and to reestablish a stable gut community^138,139^. Exposure to antibiotics can disrupt the abundance and diversity of the gut microbiota, and the introduction of deleted core microbiota can help to rebuild the gut immune homeostasis through syntrophic relationships^125^. For example, Button et al.^140^ developed a synbiotic of HMOs and *B*. *infantis* to improve the imbalance in the gut microbiota and affect the growth of *Enterobacteriaceae* by promoting the production of acetate and butyrate. A follow-up study by Button et al.^141^ found that this HMO-dependent bifidobacterial strategy could increase the abundance of lactate consumers (*Veillonella spp*.) and alleviate antibiotic-induced dysbiosis of the gut microbiota by decreasing the pro-inflammatory metabolite p-cresol sulfate.

#### Stimulating secretion of beneficial metabolites and improving metabolic status

Due to the cross-feeding relationships with butyrate producers, changes in the abundance of *Bifidobacterium* are strongly correlated with fecal acetate and butyrate concentrations, and their metabolic interactions can modulate metabolic syndromes such as type 2 diabetes (T2D) and obesity^142,143^. Butyrate plays an important role in the regulation of blood glucose levels^144^. Perraudeau et al.^145^ designed a synbiotic containing *Akkermansia muciniphila*, *B*. *infantis*, three butyrate producers, and inulin to improve T2D and determined that the cross-feeding activities of *A. muciniphila* and *B*. *infantis* with butyrate producers promoted butyrate production, which warrants in vivo validation. Multistrain synbiotics have also been used to improve energy metabolism, immune function, and gut microbiota in individuals with obesity^146,147^. Kanazawa et al.^142^ introduced a synbiotic composed of GOS, *B*. *longum*, and *B*. *bifidum* to regulate obesity, and determined that an increase in *Ruminococcus* can be used for butyrate production. Nguyen et al.^148^ used arabinoxylan to treat obesity through co-occurrence network analysis and found that *B*. *longum* released oligosaccharides through the degradation of arabinoxylan for synergistic and mutual interactions with *Bacteroides*, *Phascolarctobacterium*, and *Subdoligranulum* to promote the production of acetate and mitigate obesity. Dietary changes and synbiotic interventions can be used to increase the abundance of bifidobacteria in the gut tract and promote the secretion of beneficial metabolites, thereby mitigating metabolic disorders; however, the clinical significance remains to be determined.

##### Future perspectives

Nutrition changes, such as those from breast milk in early life to complex glycans in adulthood and beyond, directly determine the evolution of bifidobacteria in the gut. The glycan preference of bifidobacteria is useful to adapt to changes at different ages and dietary stages and introduce cross-feeding relationships between bifidobacterial strains and other gut microbiota, providing positive feedback to the host. Therefore, the glycan utilization strategies of *Bifidobacterium* and their cross-feeding networks must be explored. However, unlike *Bacteroides*, evidence of the intracellular and extracellular GHs and transport systems of *Bifidobacterium* remains scarce.

The ability of the transport system to recognize specific substrates may influence cross-feeding, which warrants further investigation. In addition, cross-feeding between *Bacteroides* and *Bifidobacterium* based on AX and AG has been reported; however, the evidence remains inadequate, and the syntrophic relationship with other glycans, such as β-glucans and fructans, remains to be elucidated. Future studies should further investigate the cross-feeding of bifidobacterial species (such as *B*. *longum* and *B*. *pseudocatenulatum*) and that between bifidobacteria and gut microbiota (such as *Lactobacillus*).

The prevalence of bifidobacteria decreases with age. The cross-feeding activities of bifidobacteria are beneficial to the host; therefore, dietary intervention strategies must be adopted to regulate health status. However, most existing studies on the health benefits of multistrain synbiotics have focused on the outcome of the intervention rather than the process and lack the scientific basis for such synergistic combinations. This necessitates the development of individual formulas and consideration of intervention times based on the gut microbiota of individuals at different ages or nutritional stages. The beneficial effects of bifidobacteria involved in multistrain synbiotics and strategies to alter the relationship between gut microbiota and host must be determined. The targeted modulation of bifidobacteria through cross-feeding strategies can shift the classic nutritional studies of flora to molecular nutrition, which will be the future trend of microbiome therapy.
